# Effects of Vaccination and the New Neuraminidase Inhibitor, Laninamivir, on Influenza Infection

**DOI:** 10.1371/journal.pone.0092601

**Published:** 2014-04-03

**Authors:** Takuro Mizuno, Shigeru Mizuno, Tsugiyasu Kanda

**Affiliations:** 1 Department of Community Medicine, Kanazawa Medical University Himi Municipal Hospital, Himi, Toyama, Japan; 2 Mizuno Clinic, Osaka, Japan; The University of Hong Kong, China

## Abstract

**Background:**

Evidence of the effectiveness of influenza vaccination in children and elderly adults is limited, although this population has the highest risk for influenza infection.

**Materials and Methods:**

We enrolled 4443 participants, aged 3–97 years, who had influenza-kit-positive resultsduring seasons 2007–12, including 2135 with influenza A, 534 with A/H1N1, and 1643 with influenza B. Eligible subjects completed a questionnaire to identify past influenza infection and vaccination history. For the diagnosis of current influenza infection, subjects were examined, and pharyngeal swabs were collected and tested using the Capilia flu rapid diagnosis kit to confirm influenza infection. An interim analysis was performed using clinician-based surveillance data for the entire four seasons of influenza infection in Japan.

**Results:**

In 3035 adultsaged 14–64 years, administration of the influenza vaccine significantly reduced the frequency of infection (P<0.01) in the 2008 and 2010 seasons, but not in the 2009 and 2011 seasons. Moreover, the vaccine did not reduce the frequency of infection in children (aged <13 years) and older adults (aged >65 years) significantly. Laninamivir, oseltamivir phosphate, zanamivir hydrate, and amantadine hydrochloride were administered to 1381, 2432, 1044, and 100 patients, respectively. They were effective in >97% of patients, with no significant differences being found. Adverse effects were few. However, the recurrence rate of influenza infection after treatment was significantly reduced in patients who received laninamivir compared with that in those who received oseltamivir and zanamivir (P<0.01). The effectiveness of laninamivirdid not decrease.

**Conclusions:**

The vaccines administered had limited efficacy in reducing the frequency of influenza infection in young adults. Laninamivir significantly reduced the recurrence of influenza infection when compared with other neuraminidase inhibitors.

## Introduction

A recent meta-analysis showed that influenza vaccination can provide moderate protection against influenza virus infection, but such protection is greatly reduced or absent in some seasons [1], [2]. A search of the Cochrane Central Register of Controlled Trials showed thatdata on influenza vaccination in healthy children and the elderlywere limited [3], [4]. Additionally, surveys have shown that decreased use of antiviral medications results in worse outcomes in seriously ill patients despite oseltamivir treatment [5]. Moreover, evidence for protection in adults aged 65 years or older is still lacking.

Patients suspected of having influenza virus infection usually present with common clinical characteristics, including fever, cough, sore throat, and arthralgia. There are conflicting reports on the effectiveness of a pharyngeal follicle swab for the diagnosisof influenza virus infection [5]. Therefore, this study examined whether these presenting factors are clinically characteristic of the early markers of an influenza virus infection and whether pharyngeal follicles could be an early and useful diagnostic tool [6].

Inhaled laninamivir was developed in Japan and approved for use in our country in 2010 [7]. Laninamiviroctanoate has been shown to have neuraminidase inhibitory activity against various influenza A and B viruses, including oseltamivir-resistant viruses [8]. The chemical structure of the active drug laninamivir is similar to that of zanamivir. The most important characteristic of laninamiviroctanoate is its long-lasting antiviral activity. As a result, laninamivir is administered as a single inhalation dose on the first day of treatment. It remains active in the respiratory tract for several days [8].

Laninamivir was more effective at rapid alleviation of influenza virus infection and associated symptoms in children with influenza A as compared to oseltamivir [9]. The decreased effectiveness of oseltamivir could be partly due to the fact that almost all seasonal A (H1N1) viruses possess the H275Y mutation, which confers resistance to oseltamivir [10].

In the present study, influenza vaccine efficacy was evaluatedin different age groups from 2007 to 2011 to investigate characteristic symptoms and the effectiveness of laninamivir as compared to other accepted treatments for influenza virus infection.

## Methods

### Study Population

An interim analysis of clinic-based surveillance data was performed, including entire data sets for four influenza seasons in Japan, to examine the effectiveness of vaccination, as well as laninamivir, in comparison with other neuraminidase inhibitors.

The study examined 4443 cases of influenza, diagnosed by quick inspection at the Mizuno Medical Clinic, from March 2007 to March 2011. Participants were treated with laninamivir, oseltamivir, zanamivir, or amantadine.

Participants were in stable health with no significant pulmonary, cardiovascular, hepatic or renal disease. Subjects were excluded if they had received any seasonal influenza vaccination within 6 months or any investigational product within 30 days prior to vaccination in this study. These cases were contacted within 7 days of visit by telephone. The interview included history of illness including cough, fever, nasal congestion, chills, or sore throat. Influenza relapse was defined as the recurrence of influenza-like symptoms.

History of influenza illness and vaccination, symptoms, and relapse rates were compared, and drug efficacy was examined. In addition, a questionnaire was administered to assess the effects of the previous season at the time of vaccination. Relapse of influenza-like symptoms was confirmed by telephone interviews with each participant one to two weeks later.

### Vaccine Strains and Epidemic Strain

Vaccines were developed and manufactured in Japan. Vaccine strains and actual epidemic strains were obtained fromthe Infectious Agents Surveillance Report [11]–[14] (Table 1).

**Table 1:** Vaccine and epidemic strains 2007–2011.

Season	H1N1	H3N2	B
Vaccine strain			
2007–08	A/Solomon Islands/3/2006	A/Hiroshima/52/2005	B/Malaysia/2506/2004
2008–09	A/Brisbane/59/2007	A/Uruguay/716/2007	B/Florida/4/2006
2009–2010	A/Brisbane/59/2007	A/Uruguay/716/2007	B/Brisbane/60/2008
2010–2011	A/California/59/2007	A/Victoria/210/2009	B/Brisbane/60/2008
Epidemic strain			
2007–08	A/Solomon Islands/3/2006* A/Brisbane/59/2007	A/Brisbane/10/2007	B/Florida/4/2006
2008–09	A/Brisbane/59/2007*	A/Uruguay/716/2007*A/Perth/16/2009	B/Bangladesh/3333/2007
2009–2010	A/California/59/2007	A/Perth/16/2009	B/Brisbane/60/2008* B/Bangladesh/3333/2007
2010–2011	A/California/59/2007*	A/Victoria/210/2009* A/Perth/16/2009	B/Brisbane/60/2008*

The virus* shows the same virus as vaccine.

### Laboratory Methods

For the diagnosis of influenza infection, throat swabs were collected and tested using the Capilia flue rapid diagnosis kit (Nippon Becton Dickinson Company Ltd., Tokyo, Japan), which utilizes immunochromatography. Physical examination, including that of pharyngeal follicles, was performed in accordance with similar recent reports [6].

### Statistical Analysis

A descriptive statistical analysis using the Chi-square and Fisher’s exact tests was performed to characterize the study subjects on the basis of whether they were vaccinated or not. Statistical analyses were performed using SAS version 9.1 for windows (SAS Institute, Cary, NC, USA). A P value of <0.05 was considered statistically significant.

### Ethics Statement

Ethical approval was obtained from the Kanazawa Medical University, Department of Community Medicine, Himi, Japan. Informed consent was obtained from each participant prior to the administration of questionnaire after the purpose of the study was explained to respondent. Confidentiality was maintained by omitting personally identifiable information, such as the participant’s name, from the questionnaire.

## Results

Almost equal numbersof men (45.8%) and women (54.2%) were enrolled. Overall, the largest proportion of participants were between the ages of 13 and 64 years (68.3%), followed by <13 years (20.9%), and >65 years (10.8%). Most participants reported a body temperature of >37°C (40%), followed by ≦38°C (35.6%) and ≦37.1°C to 37.9°C (24.5%) (Table 2). Of the total participants, 37% had influenza type B, 20.9% had influenza type A, 12% had type H1N1, and 3% had both types A and B. Among the symptoms reported, sore throat was the most common (2981 participants), followed by weakness (2874 participants), and fever (2670 participants). Other than fever, sore throat and malaise symptoms were quite prominent. Participants received oseltamivir (2432 participants), laninamivir (1381 participants), zanamivir (1044 participants), or amantadine (100 participants).

**Table 2:** Clinical characteristics of participants with seasonal influenza.

		Number (%)
Sex	Male; female	2035; 2408 (45.8;54.2)
Age	<13	929 (20.9)
	13<<64	3035(68.3)
	>65	480 (10.8)
Body temperature	≦37°C	1777 (40)
	37.1°C <<37.9°C	1085 (24.5)
	>38°C	1581 (35.6)
Type of influenza	A	2133 (48)
	B	1643(37)
	H1N1	534(12)
	A+B	133 (3)
Symptoms	Sore throat	2981 (67.1)
	Weakness	2874 (64.7)
	Fever	2670 (60.1)
	Cough	929 (20.9)
	Arthralgia	870 (19.6)
	Headache	635 (14.3)
	Abnormal behavior	278 (6.3)
	Digestive symptom	2 (0.1)
Pharyngeal follicle		3485 (78.4)

A significant difference was observed in the morbidity rates of the vaccinated participants when compared with the non-vaccinated participants in 2008/2009 and a significant difference was observed in participants between the ages of 14 and 64 years in 2010/2011 (P<0.001; Table 3). Among the participants aged <13 years or >65 years, no differences were observed between those who were vaccinated and those who were not vaccinated during these four influenza seasons.

**Table 3:** Seasonal characteristics of vaccinated and non-vaccinated patients determined using the kitsince 2007 to 2011.

Season	Age group	Vaccines	No of patients (%)	No of kit-diagnosed influenza (%)	P value
2007/8	<13	Vaccinated	162	58 (35.8)	
		Non-vaccinated	25	12 (48.0)	0.24
	14–64	Vaccinated	247	79(32.0)	
		Non-vaccinated	135	48(35.6)	0.48
	>65	Vaccinated	397	97 (24.4)	
		Non-vaccinated	54	10 (18.5)	0.34
	Total	Vaccinated	796	234(29.4)	
		Non-vaccinated	214	70(32.7)	0.26
2008/9	<13	Vaccinated	108	46 (42.6)	
		Non-vaccinated	37	16 (43.2)	0.95
	14–64	Vaccinated	175	52(29.7)	
		Non-vaccinated	142	56(39.4)	0.07
	>65	Vaccinated	375	72 (19.2)	
		Non-vaccinated	43	13 (30.2)	0.08
	Total	Vaccinated	658	170(25.8)	
		Non-vaccinated	222	85(38.3)	<0.01
2009/10	<13	Vaccinated	171	49 (28.7)	
		Non-vaccinated	32	5(15.6)	0.18
	14–64	Vaccinated	219	32(14.6)	
		Non-vaccinated	85	17(20.0)	0.09
	>65	Vaccinated	319	15 (4.7)	
		Non-vaccinated	52	0 (0)	0.11
	Total	Vaccinated	709	96(13.5)	
		Non-vaccinated	169	22(13.0)	0.86
2010/11	<13	Vaccinated	88	39 (44.3)	
		Non-vaccinated	56	27 (48.2)	0.65
	14–64	Vaccinated	231	43(18.6)	
		Non-vaccinated	171	145(84.8)	<0.001
	>65	Vaccinated	375	15 (4.0)	
		Non-vaccinated	70	3 (4.3)	0.91
	Total	Vaccinated	694	97(14.0)	
		Non-vaccinated	297	175(58.9)	<0.01

Abnormal behavior as an adverse effect occurred after administration in three participants who received oseltamivir (0.3%), one participant who received zanamivir (0.1%), and two participants who received laninamivir (0.2%). No significant differences were observed in the side effects of the different drugs, as indicated by abnormal behavior.

Laninamivir was found to be remarkably effective regardless of the progression of time after onset, body temperature at the clinic, or type of influenza (Table 4). The rate of relapse was compared forlaninamivir, oseltamivir, zanamivir, and amantadine and was much higher in participants who received oseltamivir and amantadine, than in those who received laninamivir (Table 5).

**Table 4:** Effects oflaninamivir on patients with influenza of 2011/2012 seasons.

	No of patients (n)	Effective response (n)	Effective rate (%)
Progress after the influenza onset			
<24 h	332	320	96.4
24<<48h	174	166	95.4
48h<	198	188	94.9
Total	704	674	95.7
Body temperature at the clinic			
≦37,4	390	383	98.2
37.5<<38.5	201	198	98.5
≦38.6	102	99	97.1
Total	693	680	98.1
Types of influenza			
H1N1	597	591	99
Type A	211	205	97.2
Type B	17	17	100

**Table 5:** Relapsing rate of influenza-like symptom.

	Number of patients	Relapsing number of patients	Relapsing sate (%)	P value
Laninamivir	1381	11	0.8	
Zanamivir	1044	23	2.2	NS
Oseltamivir	2432	148	6.1	0.008
Amantadine	100	12	12	0.003

## Discussion

According to the Infectious Agents Surveillance Report of the National Institute of Infectious Diseases of Japan, a pandemic influenza season occurred during 2009–2010, followed by a moderate to severe influenza season during 2007–2008 [15]. This study demonstrated that the prevalence of influenza infection did not differ between the vaccinated group and the non-vaccinated group in participants aged <13 years of age and those aged >65 years of age. Vaccine effectiveness has been reported to be lower among children and the elderly due to a diminished immune response post-vaccination [15], [16], [17], and our data showed that influenza vaccination was less effective for prevention in these populations during the 2007–2011 seasons. Furthermore, vaccination in this population may be less beneficial since adverse events occur after vaccination [18].

Epidemic influenza is known to be unpredictable worldwide [19]. The differences between vaccine and epidemic strains for the seasons 2007–08, 2008–09, and 2009–10 are shown in Table 1. Either one or twofrom H1N1, H3N2 and B influenza were mismatched in these seasons. The mismatch with the vaccine in the 2010–11 season was lowest, which may affect the significant beneficial effect in vaccinated patients aged 14 to 64 shown in Table 3.

When the rate of recurrence of influenza virus infection was analyzed, laninamivirwas not different from zanamivir but could more effectively reduce recurrence than oseltamivir and amantadine. Laninamivir was effective even when administered later than 48 h from disease onset. A significant difference in the rate of relapse was observed with laninamivir as compared with oseltamivir and amantadine. Laninamivir is effective against oseltamivir-resistant influenza virus [8], [20].

Abnormal behaviors were suspected of being adverse reactions to oseltamivir and became a cause of national concern in 2007. In March 2007, the Ministry of Health, Labour and Welfare issued an emergency to suspend the use of oseltamivir to treat patients between the ages of 10 and 19 years due to suspected abnormal behavior that was difficult to control. Delirious behavior and hallucinations have been reported in children with influenza [21], [22], and the behaviors of children after taking oseltamivir, which have been reported in Japan, may be an extension of delirium or hallucinations caused by influenza virus infection. This study did not show any significant abnormal behavior caused by these neuraminidase inhibitors.

This study also examined the effectiveness of early diagnostic tools for the identification of influenza virus infection and the clinical characteristics associated with infection. The clinical characteristics and diagnostic tools for identification of influenza virus infection were also analyzed. Typical clinical characteristics of influenza virus infection include fever, cough, myalgia, malaise, headache, sore throat, and sneezing, while the most commonly reported characteristic is fever. However, no typical fever could be identified in this study. Additionally, the most frequently reported clinical characteristic in participants was sore throat. Positive pharyngeal follicles were identified in 78% of patients with any types of influenza, which suggests that a pharyngeal follicle swab is an important early diagnostic tool for identifying influenza virus infection. The diagnostic value of pharyngeal follicle swabs for all types of influenza has been previously reported by Japanese physicians as extremely sensitive (97%–100%) [5]. However, pharyngeal examination in patients with influenza virus infection is mentioned as an unremarkable result in Harrison’s Textbook of Internal Medicine [23] despite the presence of other clinical characteristics such as sore throat. This study and previously published reports demonstrate the potential value of pharyngeal follicles as an early diagnostic tool that could be added as one of the expected clinical characteristics in patients with influenza virus infection.

This study had several limitations, including limited sample size and location. This could have influenced statistical power to detect the significance of vaccine effectiveness. Additionally, this study did not examine poor health outcomes associated with influenza virus infection such as pneumonia, hospitalization, and death, since these did not occur in any of our participants during the study period.

In conclusion, the results of this study support the limited effectiveness of influenza vaccination in children and elderly persons, despite the high risk for illness that these populations face. This study further demonstrated the clinical importance of pharyngeal follicle swabs as an early and effective diagnostic tool of influenza virus infection. Additionally, laninamivir significantly reduced the recurrence of influenza when compared with other neuraminidase inhibitors.
